# Development and validation of a combined hypoxia and ferroptosis prognostic signature for breast cancer

**DOI:** 10.3389/fonc.2023.1077342

**Published:** 2023-03-14

**Authors:** Jianxin Zhong, Xi Shen, Junjie Zhou, Heping Yu, Birong Wang, Jianbin Sun, Jing Wang, Feng Liu

**Affiliations:** ^1^Key Laboratory of Carcinogenesis and Translational Research (Ministry of Education), Department of Breast Oncology, Peking University Cancer Hospital and Institute, Beijing, China; ^2^Department of Head and Neck Oncology and Department of Radiation Oncology, Cancer Center, West China Hospital, Sichuan University, Chengdu, Sichuan, China; ^3^Department of Pathology, Union Hospital, Tongji Medical College, Huazhong University of Science and Technology, Wuhan, China; ^4^Department of Thyroid and Breast Surgery, Wuhan Fourth Hospital, Wuhan, China; ^5^Department of Thoracic Surgery, Wuhan Fourth Hospital, Wuhan, China

**Keywords:** hypoxia, ferroptosis, prognostic model, breast cancer, tumor microenvironment

## Abstract

**Background:**

Hypoxia is involved in tumor biological processes and disease progression. Ferroptosis, as a newly discovered programmed cell death process, is closely related to breast cancer (BC) occurrence and development. However, reliable prognostic signatures based on a combination of hypoxia and ferroptosis in BC have not been developed.

**Method:**

We set The Cancer Genome Atlas (TCGA) breast cancer cohort as training set and the Molecular Taxonomy of Breast Cancer International Consortium (METABRIC) BC cohort as the validation set. Least Absolute Shrinkage and Selection Operator (LASSO) and COX regression approaches were used to construct ferroptosis-related genes (FRGs) and hypoxia-related genes (HRGs) prognostic signature (HFRS). The CIBERSORT algorithm and ESTIMATE score were used to explore the relationship between HFRS and tumor immune microenvironment. Immunohistochemical staining was used to detect protein expression in tissue samples. A nomogram was developed to advance the clinical application of HFRS signature.

**Results:**

Ten ferroptosis-related genes and hypoxia-related genes were screened to construct the HFRS prognostic signature in TCGA BC cohort, and the predictive capacity was verified in METABRIC BC cohort. BC patients with high-HFRS had shorter survival time, higher tumor stage, and a higher rate of positive lymph node. Moreover, high HFRS was associated with high hypoxia, ferroptosis, and immunosuppression status. A nomogram that was constructed with age, stage, and HFRS signature showed a strong prognostic capability to predict overall survival (OS) for BC patients.

**Conclusion:**

We developed a novel prognostic model with hypoxia and ferroptosis-related genes to predict OS, and characterize the immune microenvironment of BC patients, which might provide new cures for clinical decision-making and individual treatment of BC patients.

## 1 Introduction

Breast cancer has been the most prevalent tumor in women worldwide and is the leading cause of cancer-related death among women with malignant diseases (1, 2). With the advancement of diagnosis and treatment, the overall survival of primary breast cancer has been greatly improved; however, advanced breast cancer is still refractory, and some patients who were diagnosed with distant metastasis that lose the chance of surgery therapy were required more efficient target drugs to improve their prognosis. Breast cancer is a heterogeneous tumor; individual treatment and biological feature depiction of each patient is a field that calls for exploration (3). Although age at diagnosis, tumor stage, and histological grade are considered as prognostic factors, there are few reliable biomarkers based on personal gene expression pattern to facilitate clinical assessment (4–7). Therefore, it is important to discover novel prognostic factors and potential therapeutic targets for individual treatment.

Hypoxia is a feature of solid tumors generated since the supply could not meet the consumption of oxygen under rapid tumor proliferation, and form a tumor microenvironment (8, 9). Hypoxia could induce tumor angiogenesis, cell proliferation, metastasis, and invasion and promote tumor immune suppression and escape, while reducing apoptosis, differentiation, and ferroptosis to accelerate tumor progression (8, 10–12). Ferroptosis, as a newly discovered regulatory cell death, was closely related to tumor development (13, 14). Increasing evidence revealed that targeting ferroptosis induced treatment response in BC (15–17). More importantly, hypoxia has been proven to participate in the regulation of ferroptosis (18, 19). Some studies demonstrated that hypoxia blocks ferroptosis in hepatocellular carcinoma, and HIF-1α-induced lncRNA PMAN promoted gastric cancer peritoneal dissemination by inhibiting ferroptosis (20, 21). However, the association between hypoxia and ferroptosis in BC has not been reported yet.

Immune microenvironment regulation was critical in tumor progression, which has been wildly verified to be associated with hypoxia status (22, 23). Some studies have reported that hypoxia inducible factor-1α (HIF-1α) increased PD-L1 expression and antigen non-specific T-cell suppression, and promote the differentiation of MDSC to immune suppressive TAM in various kinds of tumors including breast cancer (24–27). HIF-1α could negatively regulate the functions of CD4+ and CD8+ T lymphocytes, and depletion of HIF-1α enhanced T cell response (28, 29). Interestingly, many evidences indicated that ferroptosis was also involved in the regulation of the immune microenvironment and immunotherapy resistance in cancers (30–33). Thus, there was potential interaction between hypoxia and ferroptosis, and either of them was associated with immune microenvironment regulation in cancers.

Given that hypoxia and ferroptosis are related to breast cancer prognosis, there were few studies that reported the crosstalk between hypoxia and ferroptosis, and no prognostic signature has been established in BC for risk stratification and immune microenvironment profiling. This study firstly combined ferroptosis-related genes (FRGs) with hypoxia related genes (HRGs) to construct a prognostic signature HFRS to predict BC prognosis and immune status.

## 2 Materials and methods

### 2.1 Data acquisition

The mRNA expression data and corresponding clinicopathological information of BC patients were obtained from the TCGA and METABRIC websites. A total of 1075 BC patients from the TCGA database were enrolled in the training cohort, and 1399 patients with completed clinical information from the METABRIC database were included as a validation cohort, after excluding patients who lacked tumor stage and survival information. By intersecting the ferroptosis-related genes in the FerrDb database and Molecular Signatures Database3, 47 FRGs were retrieved; 243 HRGs were downloaded from the hypoxia-related gene set “winter_hypoxia_metagenes” in Molecular Signatures Database 3 (MSigDB: https://www.gsea-msigdb.org/gsea/msigdb). Gene expression data from these databases were normalized by the R package “limma”. **Supplementary Table 1** shows the clinicopathological information of TCGA and METABRIC cohort in this study. Identification of PAM50 subtypes of all the patients was performed by the ‘genefu’ R package based on gene expression profiles.

### 2.2 Development of the HFRS

Univariate COX regression analysis was used to screen prognostic genes among 47 FRGs and 243 HRGs in the TCGA cohort. Then, 15 FRGs and HRGs significantly associated with prognosis identified in univariate regression analysis (p< 0.001) of BC patients were input into the Least Absolute Shrinkage and Selection Operator (LASSO) The COX regression model was used to identify the critical genes and the corresponding regression coefficient by using the R package “glmnet” (Friedman et al., 2010). We constructed a hypoxia and ferroptosis prognostic signature (HFRS) for the BC patients with 10 FRGs and HRGs selected by LASSO COX analysis. HFRS scores were calculated for all patients according to the formula: lambda.min = 0.0027


$$\begin{array}{l} \text{Risk score}=\left(-0.1875\right)*\text{BTG}1+\left(-0.2695\right)*\text{CCT}6\text{A}+\left(-0.033\right)*\text{KRT}14+\\ \left(0.1338\right)*\text{P}4\text{HA}2+\left(0.431\right)*\text{PGK}1+\left(0.1802\right)*\text{SLC}16\text{A}2+\left(-0.0571\right)*\text{STC}2+\\ \left(-0.0268\right)*\text{TF}+\left(0.0715\right)*\text{TPD}52+\left(0.3014\right)*\text{CISD}1: \end{array}$$

$$HFPS=\sum_{i=1}^{n}coef_{i}*x_{i}$$

Where *xi* is the expression level of each FRG or HRG and *Coefi* is the coefficient.

Then the R package “survminer” was used to calculate the optimal cut-off value (this is an outcome-oriented method providing a value of a cut-off point that corresponds to the most significant relation with survival) and the patients were divided into two subgroups (low-HFPS and high-HFRS group) according to the optimal cut-off value.

### 2.3 Functional analysis

Gene Set Enrichment Analysis (GSEA) was used to investigate the pathways enriched in the low-HFRS subgroup and high-HFRS subgroup and identified significant enrichment pathways with normalized enrichment score >1, nominal p< 0.05, and false discovery rate q< 0.25. Differentially expressed genes (DEGs) between the high HFRS and low-HFRS groups were obtained using the R package “Deseq2” (| log2(Fold change) |>1 and adjust p<0.05) and were input into “ClusterProfiler” R package for functional enrichment and pathway analysis, including the Kyoto Encyclopedia of Genes and Genomes Pathway (KEGG pathway) and Gene Ontology (GO) analysis. The FRGs and HRGs significantly associated with the prognosis (p< 0.001) of BC patients were subjected to construct a protein–protein interaction (PPI) network by MetaScape (https://metascape.org/).

### 2.4 Analysis of immune cell infiltration

To investigate the difference in immune infiltration status between patients in the high- and low-HFRS group, the CIBERSORT algorithm was used to analyze the immune cell type-specific gene expression profiles of BC patients with the LM22 signatures. Moreover, we also used the ESTIMATE method to calculate immune cell characteristics for BC patients. We downloaded the Immunophenoscores (IPS) of BC patients of the TCGA cohort from the TCIA database (https://tcia.at/) to predict the sensitivity of immune therapy of the high- and low-HFRS groups.

### 2.5 Analysis of genetic alteration in BC patients

The R package “Maftools” was used to visualize the single nucleotide variation (SNV) profile of TCGA BC patients with mutation data, and compared the different mutation patterns between the high- and low-HFRS groups. The copy number variation (CNV) of HFRS genes and their correlation with mRNA expression were analyzed in the GSCAlite (http://bioinfo.life.hust.edu.cn/web/GSCALite/) website.

### 2.6 Immunohistochemistry staining of HFRS gene protein expression in tissues

We collected 20 pairs of BC tissues and adjacent normal breast tissues from Wuhan Pu-Ai Hospital, which was approved by the ethics committees of Pu-Ai Hospital (No. KY2022-050-02). The BC tissues and adjacent normal breast tissues were fixed with 10% formalin, embedded by paraffin, and sectioned; then we selected the optimal tissue sections for degreasing and immunohistochemistry staining. Protein expression levels were evaluated semi-quantitatively following the Allred scoring system guidelines and scored separately by two qualified pathologists (34). Then, the sections were scanned to obtain high-resolution digital images using a 3DHISTECH scanner (Pannoramic, TaiBei). Antibodies used in this study are as follows: BTG1 (Proteintech, Cat No. 14879-1-AP). SLC16A2 (Abcam, ab192828). In addition, immunohistochemical staining images of the remaining eight HFRS genes were obtained from the Human Protein Atlas (HPA).

### 2.7 Statistical analysis

In this study, all statistical analysis was conducted by R 4.1.1. Univariate COX regression was used to identify independent prognostic risk factors. Multivariate COX regression analysis was used to construct a nomogram to predict OS for BC patients. The predictive efficiency of the nomogram was verified in METABRIC cohorts. The R package ‘rms’ was used in the construction and validation of the nomogram. In addition, The ROC curve and AUC were used to analyze the prognosis predictive accuracy of nomogram and other prognostic factors *via* R package “timeROC”. For descriptive statistics, mean ± standard deviation (SD) or median (range) was used for continuous variables; Student’s *t*-test and Mann–Whitney *U* test were used to analyze the difference between two groups of continuous variables. Fisher exact test or Wilcoxon’s test was used to compare the difference of clinical features of categorical variables between two groups when appropriate. Two-tailed p< 0.05 was considered as statistically significant.

## 3 Results

### 3.1 Construction of the HFRS in the TCGA cohort

**Figure 1:**
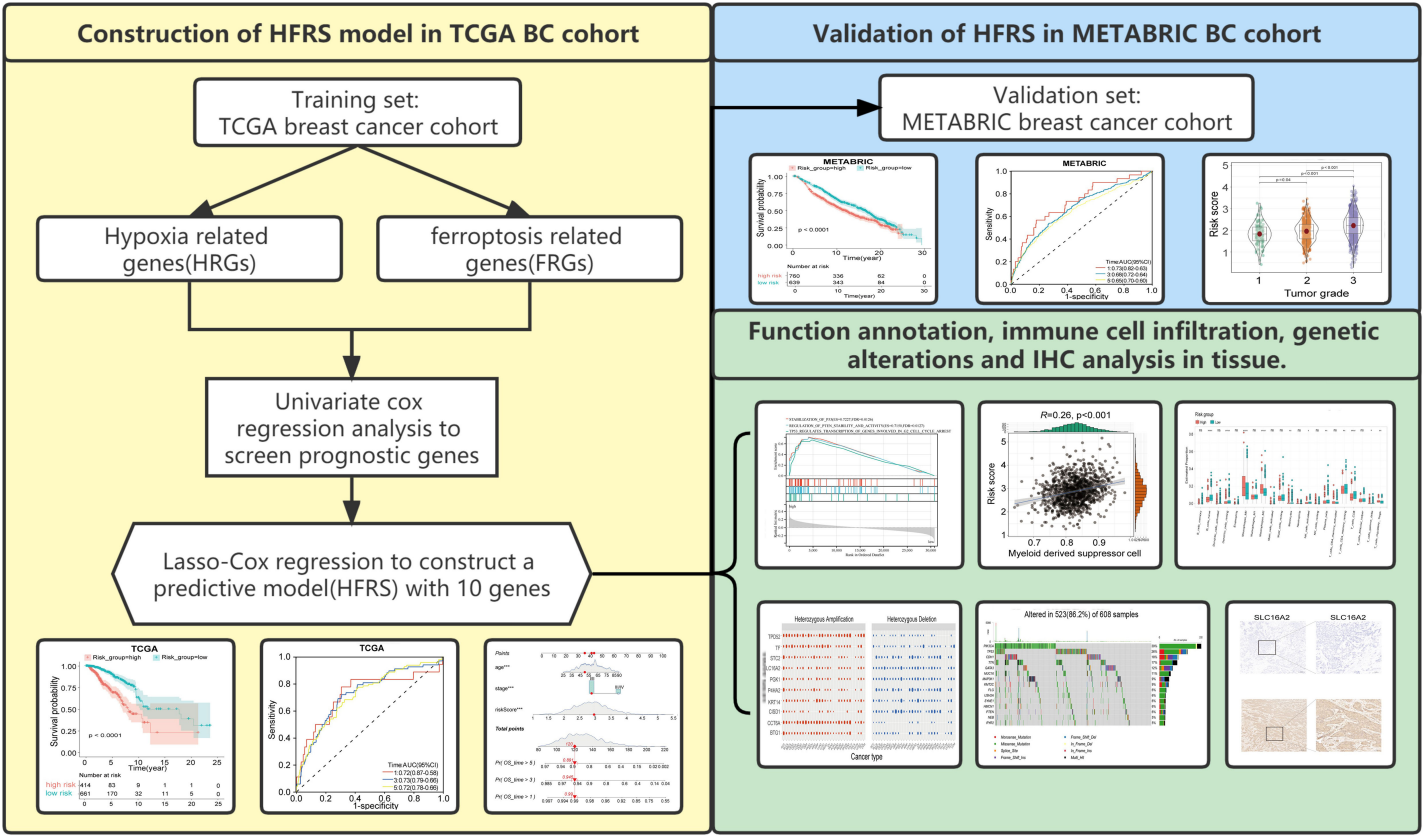
Study flowchart.

**Figure 2:**
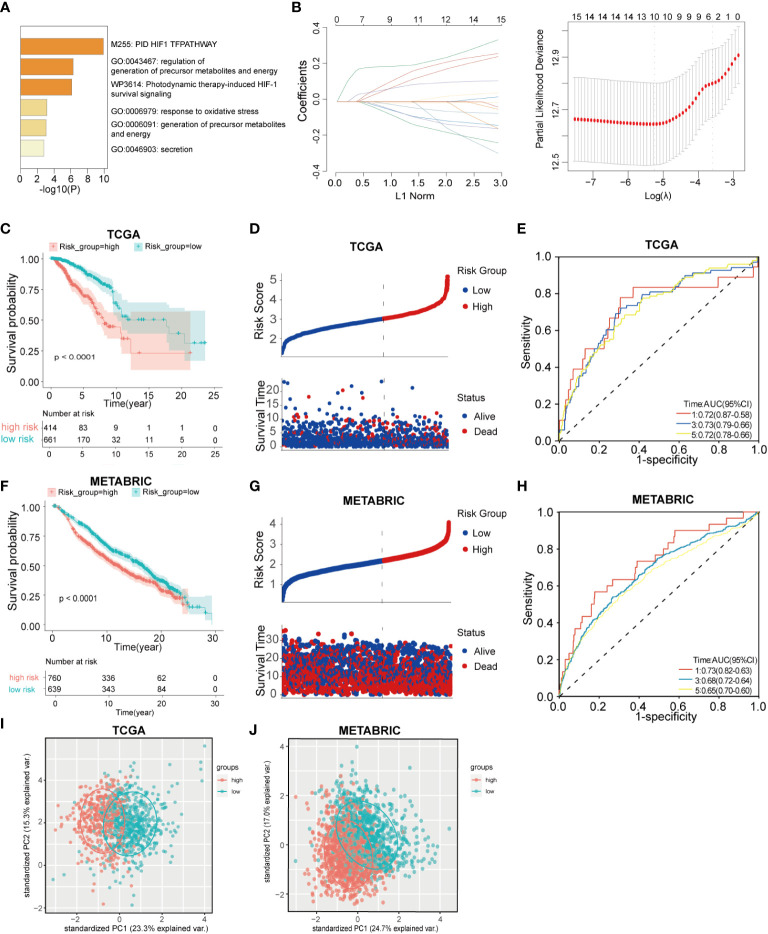
Construction and validation of hypoxia and ferroptosis-related gene risk signature(HFRS) in BC patients. **(A)** The barplot shows the enrichment of Go pathways significantly associated with prognostic HRGs and FRGs. **(B)** LASSO regression analysis identified 10 hub genes to construct HFRS signature. **(C)** Kaplan–Meier curves show the significant difference in overall survival between high- and low-HFRS groups in TCGA cohort. **(D)** The ranked dot plot indicates the HFRS_score distribution; scatter plot present the patients’ survival status in TCGA cohort. **(E)** The ROC curves of HFRS for predicting 1, 3, and 5 years overall survival in TGCA cohort. **(F)** The K-M curves show the significant difference in overall survival between high- and low-HFRS groups in the METABRIC cohort. **(G)** The ranked dot plot indicates the HFRS_score distribution; scatter plot presents the patients’ survival status in the METABRIC cohort **(H)** The ROC curves of HFRS for predicting 1, 3, and 5 years overall survival in METABRIC cohort. **(I)** The PCA plot based on HFRS gene expression show the distinct subgroups of TCGA BC cohort. **(J)** The PCA plot based on HFRS gene expression divided the METABRIC BC cohort to two subgroups.

The study design is shown in the flow chart (**Figure 1**), The GO pathways analysis conducted in the Metascape website showed that these genes were enriched in hypoxia, metabolites, energy, and oxidative stress-related pathways (**Figure 2A**). Univariate COX regression was used to screen for hypoxia-related prognostic genes (HRGs) and ferroptosis-related prognostic genes (FRGs) in the TCGA cohort. In the condition of p< 0.001, there were 15 prognostic HRGs and FRGs that were significantly associated with the prognosis of BC patients (**Supplementary Table 2**). Subsequently, 15 prognostic FRGs and HRGs were subjected to the LASSO-Cox regression analysis, and we screened 10 genes (BTG1, CCT6A, KRT14, P4HA2, PGK1, SLC16A2, TPD52 and STC2 as HRGs, CISD1 as FRGs, and TF as both HRG and FRG) to construct a hypoxia and ferroptosis prognostic combined signature (HFRS) (**Figure 2B**). HFRS scored the BC patients in TCGA cohort and the patients were further divided into the high-HFRS group (n = 414) and low-HFRS group (n = 661) according to the optimal cut-off value. Kaplan–Meier curves and log-rank test showed that patients in the high-HFRS group had a significantly worse prognosis than the low-HFRS group (p< 0.001) (**Figure 2C**). The distributions of the survival status and HFRS score are shown in **Figure 2D**. The ROC curves indicated an efficient prognostic predictive capacity of HFRS for the overall survival of BC patients; the AUC of 1, 3, and 5 years were 0.72, 0.73, and 0.72, respectively (**Figure 2E**).

### 3.2 Validation of the HFRS in the METABRIC cohort

HFRS scores of BC patients in the METABRIC cohort were calculated by the same signature model, and the patients were divided into the low-HFRS group (n = 639) and high-HFRS group (n = 760) according to the optimal cut-off value. The results of the METABRIC cohort are generally consistent with those of the TCGA cohort; patients in the high-HFRS group had significantly poorer prognosis (**Figure 2F**). The distribution of survival status and HFRS score also indicated that patients with higher HFRS scores had shorter overall survival time and higher mortality (**Figure 2G**). The ROC curve showed that HFRS score also had strong predictive power in the METABRIC cohort. The AUCs were: 0.73 (1 year), 0.68 (2 years), and 0.65 (3 years) (**Figure 2H**). In addition, the PCA suggested that the BC patients could be distinctively clustered by PCA according to HFRS as well (**Figures 2I, J**).

### 3.3 Prognostic analysis and genetic alteration of the 10 FHRS genes

**Figure 3:**
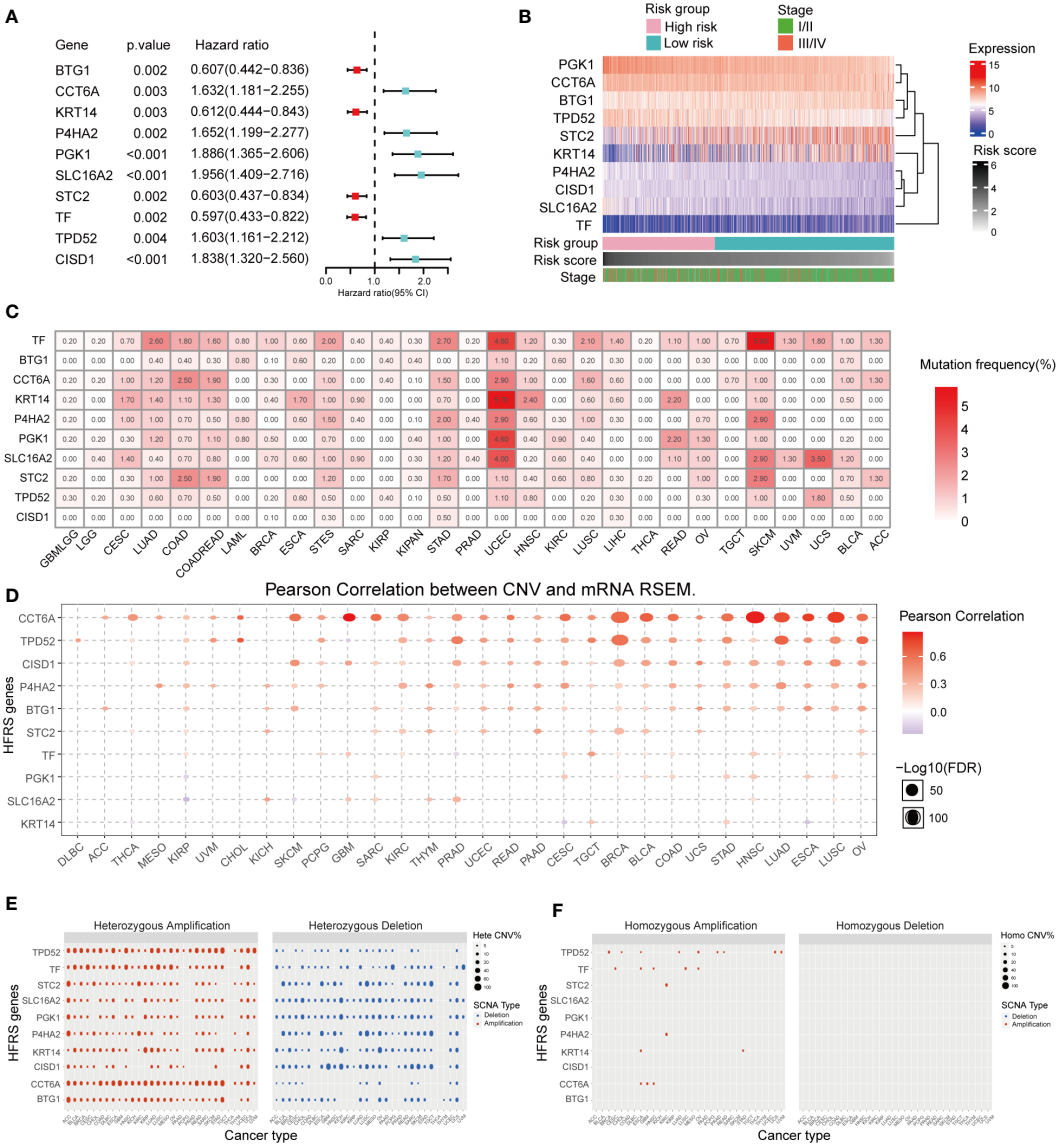
Prognosis value and expression of HFRS hub genes. **(A)** Forest plot shows the prognostic value of 10 prognostic genes in signature. **(B)** Heat map shows the relationship between mRNA expression levels of 10 HFRS genes, HFRS score, and tumor stage features in the TCGA cohort. **(C)** The mutation frequencies of 10 HFRS genes in pan-cancer. **(D)** The correlation between CNV and mRNA expression of HFRS genes in pan-cancer using Pearson analysis. The size of bubble indicated the -log10 (FDR) value. **(E, F)** The profiles of heterozygous **(E)** and homozygous **(F)** amplification/deletion of 10 HFRS genes in cancers.

Univariate COX regression analysis of 10 HFRS genes showed that BTG1, KRT14, STC2, and TF were protective factors in BC (0< Hazard Ratio (HR)< 1; p< 0.001), while CCT6A, P4HA2, PGK1, TPD52, SLC16A2, and CISD1 were risk factors (HR > 1; p< 0.001) for the overall survival of BC patients (**Figure 3A**). In addition, the heat map shows the differential expressions of 10 HFRS genes in TCGA breast cancer samples. The expressions of PGK1, CCT6A, P4HA2, TPD52, SLC16A2, and CISD1 increased with the HFRS scores while the expression of BTG1, KRT14, STC2, and TF decreased with the HFRS scores. In addition, the distribution of HFRS gene expressions was also associated with the tumor stage of BC patients (**Figure 3B**). We further explored the genetic alteration of HFRS genes in cancers. We investigated the single nucleotide variations (SNVs) of HFRS genes in different cancers and observed that some genes (CCT6A, TF, KRT14, P4HA2, PGK1, SLC16A2, and STC2) were frequently mutated in COAD, STAD, UCEC, and SKCM (**Figure 3C**). In BRCA, BLCA, HNSC, and LUAD, the copy number variations (CNVs) of some genes were positively correlated with mRNA level (**Figure 3D**). Moreover, we found that TPD52, TF, and CCT6A were more frequently heterozygous amplified while STC2, CISD1, and P4HA2 were more likely to occur in heterozygous deletion in cancers (**Figure 3E**). In contrast, homozygous amplification and deletion of HFRS genes were very rare in cancers (**Figure 3F**).

### 3.4 Clinical effects of HFRS on breast cancer patients

**Figure 4:**
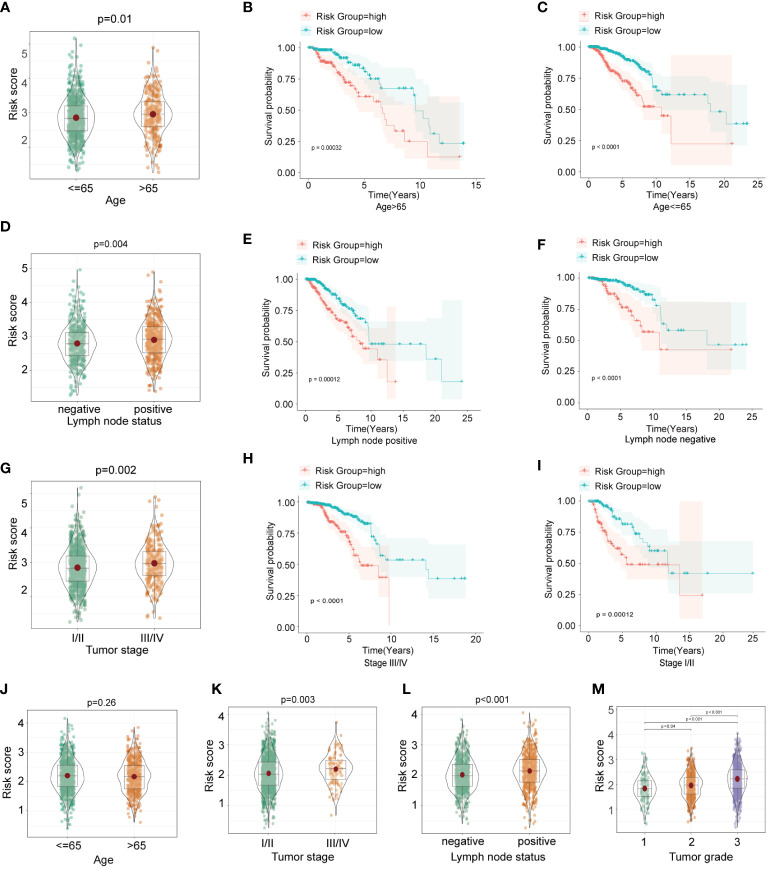
The relationship between HFRS score and clinicopathological features in BC patients. **(A, D, G)** The boxplots shows the comparison of HFRS risk score of BC subgroups stratified with different clinicopathological features (age>65 or<=65; positive or negative lymph node status; III/IV or I/II tumor stage) in TCGA cohort. **(B, C)** Comparison of the overall survival of patients with high- and low- HFRS risk score in age>65 **(B)** and age<=65 **(C)** subgroups. **(E, F)** KM curves to show the different overall survival of patients with high or low HFRS risk scores in lymph node positive **(E)** and lymph node negative **(F)** subgroups. **(H, I)** Comparison the survival of patients with high or low HFRS risk score in tumor stage III/IV **(H)** and tumor stage I/II **(I)** subgroups. **(J–M)** The boxplots shows the significant difference HFRS risk score levels of patients with different clinicopathological features(age, tumor stage, lymph node status and primary tumor grade) in METABRIC cohort.

To investigate whether the HFRS score was associated with clinicopathological characteristics of BC patients, we compared the HFRS score of BC patients according to different clinical subgroups such as BC patients of age >65; positive lymph node status and TNM stage III/IV had significantly higher HFRS scores. The result suggested that HFRS related to clinical features of BC patients, and might reflect tumor burden (**Figures 4A, D, G**). In addition, we identified overall survival in different clinical subgroups using Kaplan–Meier curves. The result suggested that BC patients with low HFRS had better OS than the patients with high HFRS in both age >65 and ≤65 subgroups (**Figures 4B, C**), and the same results were observed in negative and positive lymph node subgroups (**Figures 4E, F**), stage I/II, and III/IV subgroups (**Figures 4H, I**). Moreover, except for the age subgroup (p = 0.26), the similar HFRS distribution in these subgroups was observed in the subgroups of the METABRIC BC cohort (**Figures 4J–L**). In addition, we found that patients with higher tumor grade had significantly higher HFRS (**Figure 4M**). These results indicated that the HFRS was an effective signature to predict prognosis and was associated with BC clinical characteristics.

### 3.5 Analysis of ferroptosis and hypoxia status

**Figure 5:**
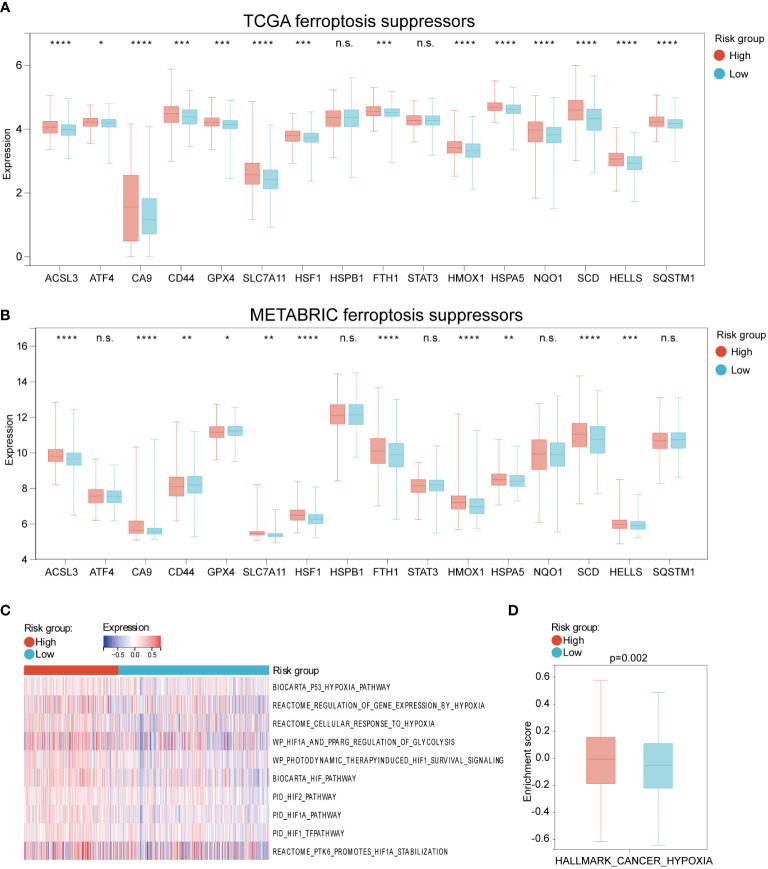
The different expression of ferroptosis and hypoxia regulations between high and low HFRS risk groups. **(A, B)** The boxplots shows the difference in ferroptosis suppressors mRNA expression between the high- and low-HFRS groups of the TCGA **(A)** and METABRIC **(B)** cohorts. **(C)** The heat map shows the association between Reactome hypoxia-related pathways and HFRS score in TCGA BC samples. **(D)** The boxplot shows the significant difference in the enrichment score of HALLMARK_CANCER_HYPOXIA between high- and low-HFRS risk groups. (*p< 0.05; **p< 0.01; ***p< 0.001; ****p< 0.0001, n.s., not significant).

To explore whether HFRS could assess the ferroptosis status of BC patients, we first compared the expression of ferroptosis suppressors and drivers in high and low-HFRS groups. As shown in **Figure 5A**, in the TCGA cohort, except for STAT3 and HSPB1, the expression of the rest ferroptosis suppressors (ACSL3, ATF4, CA9, CD44, FTH1, GPX4, HELLS, HMOX1, HSF1, HSPA5, HSPB1, NQO1, SCD, SLC7A11) was significantly higher in the high-HFRS group (**Figure 5A**). To validate the result, we also analyzed the expression of the above genes in the METABRIC cohort, and the result was similar to those of the TCGA cohort (**Figure 5B**). In addition, we also compared the expression of ferroptosis drivers between the two groups in the TCGA cohort and METABRIC cohort. The results showed that in the low-HFRS group, the expression of more than half of the ferroptosis drivers (ALOX12 ANO6, ATF3, ATG5, ATG7, EGFR, CHAC1, EGLN2, ELAVL1, IREB2, KEAP1, NCOA4, and VDAC2 in TCGA cohort; ALOX12, ALOX15, ANO6, ATM, BAP1, DPP4, EGLN2, ELAVL1, IDH1, IREB2, KEAP1, NCOA4, SAT1, and VDAC1 in the METABRIC cohort) were significantly higher than in the high-HFRS group (**Supplementary Figures 1A, B**). These results suggested that ferroptosis might be induced in patients of the low-HFRS group.

We further explored the hypoxia status of BC patients in the TCGA cohort by using GSEA analysis to estimate the enrichment scores of hypoxia and hypoxia-induced factors (HIFs) signal-related gene sets from REACTOME website. As shown in **Figure 5C**, these gene sets were enriched in the high-HFRS group, indicating that hypoxia status may be induced in the high-HFRS group. Furthermore, the box gram shows that the HALLMARK_CANCER_HYPOXIA enrichment score was significantly higher in high-HFRS group than low-HFRS group (p< 0.05) (**Figure 5D**). These results also implied that BC patients with high HFRS exhibit high hypoxia status.

### 3.6 Analysis of tumor immune cell infiltration

**Figure 6:**
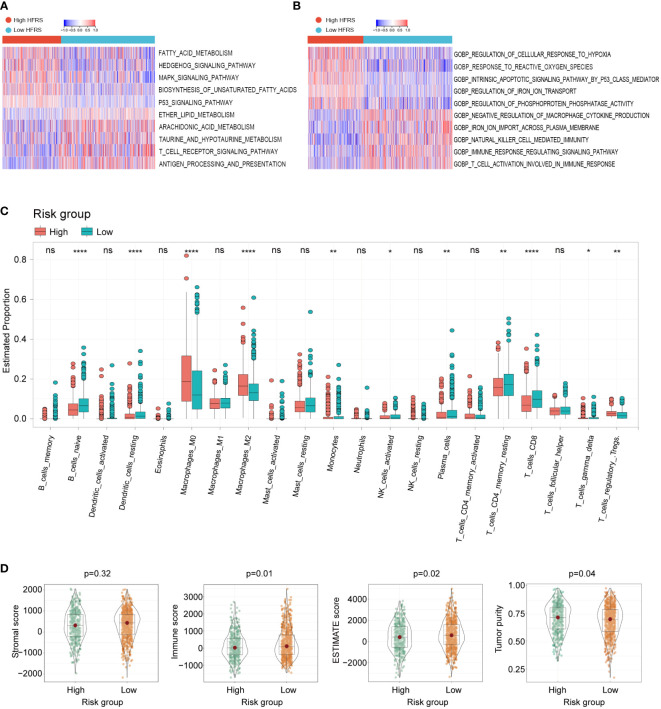
The relationship between tumor immune cell infiltration and HFRS in BC patients. **(A, B)** KEGG analyses and GO analyses for hypoxia and ferroptosis-related genes of the high- and low-HFRS groups. **(C)** Comparison of the immune cells infiltration between the high- and low-HFRS groups of the TCGA cohort by the CIBERSORT algorithm. **(D)** The violin plots show significant difference in stromal, immune ESTIMATE scores and tumor purity of between high- and low-HFRS risk groups in TCGA cohort. (*p< 0.05; **p< 0.01; ****p< 0.0001, n.s., not significant).

To investigate whether HFRS was associated with tumor immune microenvironment, the GSVA was used to analyze the enrichment of KEGG and GO pathways in high-HFRS and low-HFRS groups. KEGG analysis revealed that the following pathways were significantly activated in the high-HFRS group: fatty acid metabolism, HEDGEHOG signaling pathway, MAPK signaling pathway, TP53 signaling pathway, and others. The T-cell receptor signaling pathway, antigen processing, and presentation were significantly enriched in the low-HFRS group (**Figure 6A**). In addition, gene ontology (GO) biopathway analysis revealed that hypoxia and ferroptosis genes in low-HFRS group were significantly enriched in the immune-related functional sets such as natural killer cell-mediated immunity, T cell activation involved in immune response, and immune response regulating signaling pathway (**Figure 6B**). Thus, besides reflecting hypoxia and ferroptosis status, the HFRS might also be related to the tumor immune microenvironment.

We further investigated the difference in tumor immune cell infiltration between low- and high-HFRS patients. In CIBERSORT analysis, the fraction of B_cells_naive, Monocytes, NK_cells_activated, T_cells_CD4_memory_resting, T_cells_CD8, and T_cells_gamma_delta was significantly higher in the low-HFRS group, while Macrophages_M0/M2 and Tregs were significantly lower in the TCGA BC cohort (**Figure 6C**). The different infiltration fraction of 28 immune cells in the high- and low-HFRS groups was compared by ssGSEA as well (**Supplementary Figure 2**). Results of ssGSEA were generally consistent with those of CIBERSORT analysis. For instance, Activated_B_cell, Natural_Killer_cell, Activated_CD8_T_cells, and Monocyte were significantly highly infiltrated in samples of the low-HFRS group, while the Regulatory_T_cell was highly infiltrated in high-HFRS BC samples. Moreover, high infiltration of myeliod-derived suppressor cell and Immature_dendritic_cell and a lower Mast_cell were detected in the high-HFRS group by ssGSEA. These data revealed that the HFRS score was associated with immune cell infiltration in breast cancer.

In addition, the results of the ESTIMATE analysis showed that the immune score and tumor purity of the low-HFRS group were significantly higher than in the high-HFRS group, while the ESTIMATE score was lower than in the high-HFRS group (**Figure 6D**). Moreover, the IPS scores of the low-HFRS group were significantly higher than in the high-HFRS group in all the four subgroups (ips_ctla4_neg_pd1_neg, ips_ctla4_neg_pd1_pos, ips_ctla4_pos_pd1_neg, ips_ctla4_pos_pd1_pos) (**Supplementary Figure 3**). Thus, these results revealed that high HFRS might be associated with reduced anti-tumor immunity and decreased tumor purity.

### 3.7 Analysis of pathway and process enrichment

We identified a total of 272 DEGs between the high-HFRS group and the low-HFRS group with the criteria | log2(fold change) | > 1 and p< 0.05 (**Supplementary Figure 4A**). Then, DEGs were subjected to GSEA analysis based on REACTOME gene sets. The results showed that the DEGs were significantly enriched in the following terms: cell cycle and cellular response to hypoxia (**Supplementary Figures 4B, C**). Moreover, several tumor-related pathways and metabolism-related gene sets were enriched in the high-HFRS subgroup, such as CHOLESTEROL_HOMEOSTASIS, MTORC1_SIGNALING, TP53-PTEN related gene sets, MYC targets V1, E2F_TARGETS, PIK3-AKT-MTOR signaling (**Supplementary Figures 4D, E–H**). These results suggested that HFRS might be related to multiple tumor biology processes *via* communicating with cell cycle regulation, hypoxia microenvironment, energy metabolism, and oncogenic signal pathways, and may provide a new perspective and help us to find the potential therapeutic targets from cancer-related pathways.

### 3.8 Analysis of the gene mutation profile of different HFRS groups

**Figure 7:**
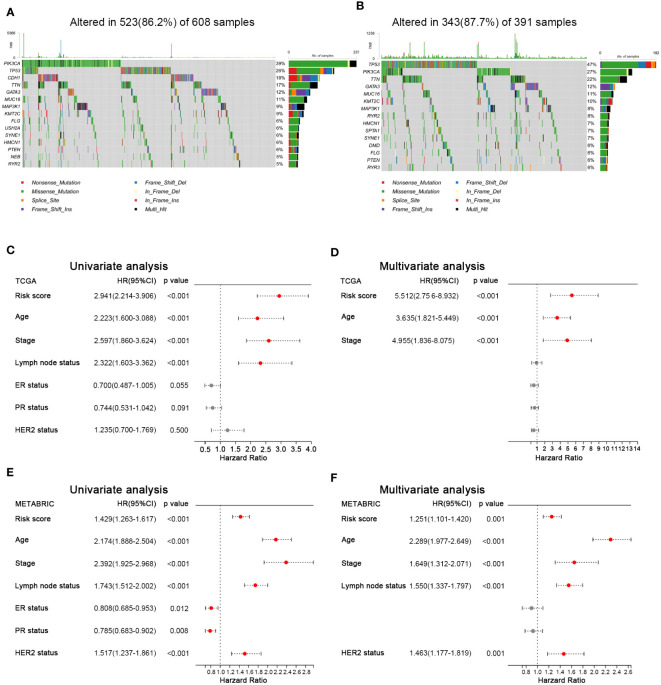
Mutation landscape and prognostic factors of BC patients. **(A, B)** Oncoplots show the mutated genes in the high-HFRS **(A)** and low-HFRS **(B)** groups of the TCGA cohort. **(C)** Forrest plots of univariate and multivariate analyses show the independent prognostic predictors in the TCGA and METABRIC cohorts.

To investigate the difference of gene mutation between the high- and low-HFRS groups, we analyzed the simple nucleoside variation profile of two groups in the TCGA cohort. As shown in **Figures 7A, B**, in the low-HFRS group, the top five genes with mutation frequency were PIK3CA (39%), TP53 (26%), CDH1 (18%), TTN (13%), and GATA3 (12%), while those in the high-HFRS group were TP53 (47%), PIK3CA (27%), TTN (22%), GATA3 (12%), and MUC16 (11%). TP53 is one of the most important tumor suppressor genes, whose mutation could lead to tumor occurrence and progression, and might be associated with suppressed ferroptosis and anti-tumor immunity (35, 36). TP53 mutation indicated worse prognosis in breast cancer (37). In this study, we found that the mutation frequency of TP53 in high HFRS was higher than in low HFRS (47% *vs*. 26%), which also suggested that high HFRS might be associated with TP53 mutation-induced prognostic risk.

### 3.9 HFRS is an independent prognostic factor for BC

To identify the clinical factors to predict the prognosis in BC, we used univariate and multivariate Cox regression analysis to estimate the hazard ratio with HFRS score and other clinicopathological features in both cohorts. The results of univariate Cox regression analysis showed that HFRS was a strong risk factor for OS in BC patients (in the TCGA cohort, HR: 2.941, 95% confidence interval (CI): 2.214–3.906, p< 0.001; in the METABRIC cohort, HR: 1.429, 95% CI:1.263–1.617, p< 0.001; **Figures 7C, E**). The multivariate regression analysis showed that HFRS was an independent prognostic factor for BC patients (in TCGA, HR: 5.512, 95% CI: 2.756–8.932, p< 0.001; In METABRIC, HR: 1.251, 95% CI:1.101–1.420, p< 0.001; **Figures 7D, F**). Then, the survival analysis of DFS and RFS of BC patients showed that patients with higher HFRS score have significantly shorter DFS and RFS (**Supplementary Figure 5**). The above results indicated that HFRS was an independent prognostic factor for BC patients.

### 3.10 Construction and validation of nomogram base on HFRS

**Figure 8:**
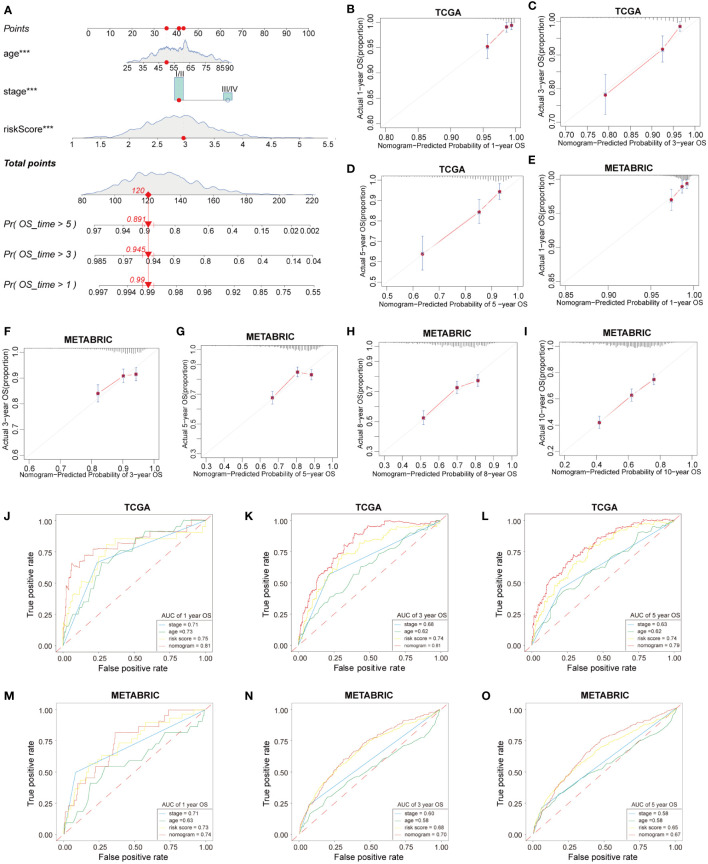
Construction and validation of nomogram. **(A)** The nomogram based on HFRS risk score, age and tumor stage for predicting overall survival of BC patients. **(B–D)** Calibration plots of the nomogram for predicting the probability of OS at 1, 3, and 5 years in the TCGA cohort. **(E-I)** The calibration plots of the nomogram for predicting 1-, 3-, 5-, 8-, and 10-year OS in the METABRIC cohort. **(J–L)** ROC curves of nomogram, risk score, tumor stage, age for predicting 1-, 3-, 5-year OS in the TCGA cohort. **(M–O)** ROC curves of nomogram, risk score, tumor stage, age for predicting 1-, 3-, 5-year OS in the METABRIC cohort.

We developed a nomogram based on HFRS and other independent prognostic factors (TNM stage) in the TCGA cohort to affiliate the application of HFRS in clinical practice (**Figure 8A**), which was validated in the METABRIC cohort. Calibration curves show that the predicted rates were highly concordant with the actual rates for 1-, 3-, and 5-year survival in the TCGA cohort (**Figures 8B–D**), and 1-, 3-, 5-, 8- and 10-year survival in the METABRIC cohort (**Figures 8E–I**). Moreover, ROC curves show that the prognostic predictive ability of the nomogram model in BC patients was better than other factors (including HFRS score, age, and TNM stage). The AUCs of 1, 3, and 5 years reached 0.81, 0.81, and 0.79 in TCGA cohort (**Figures 8J–L**) and 0.74, 0.70, and 0.67 (1, 3, and 5 years) in the METABRIC cohort (**Figures 8M–O**). These results indicated that the nomogram, based on HFRS score and TNM stage, has a strong and stable ability to predict the OS of BC patients.

### 3.11 Protein expression of 10 HFRS genes in normal breast tissues and BC tissues

**Figure 9:**
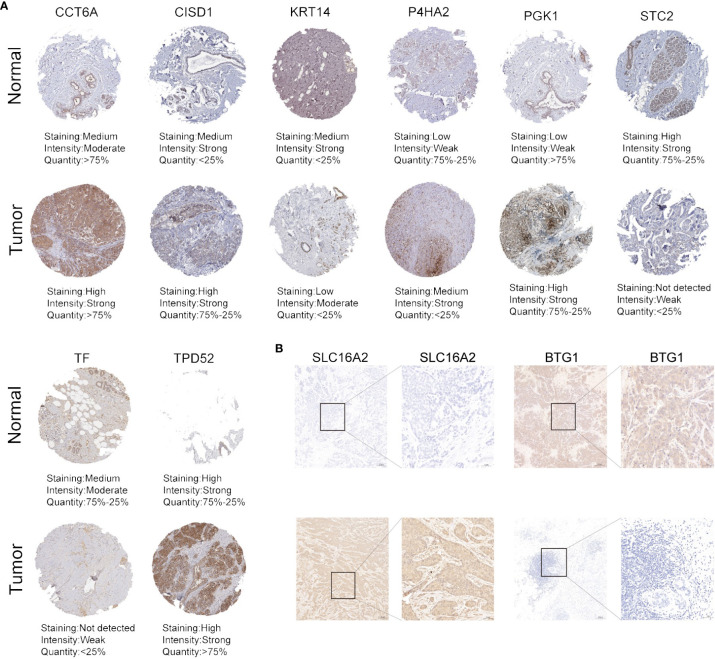
The protein expression of HFRS genes in normal and tumor samples. **(A)** Representative immunohistochemistry images of CCT6A, CISD, KRT14, P4HA2, PGK1, STC2, TF, and TPD52 expression between BC tissues and adjacent normal tissues. **(B)** IHC staining shows the protein expression of SLC16A2 and BTG1 in BC tissues and adjacent normal tissues. Scale bar: 100μm.

To explore the protein expression of HFRS genes in BC tumor tissues and normal breast tissues, we first collected the immunohistochemical staining images of several HFRS genes from HPA; the protein expression of CCT6A, CISD1, P4HA2, PGK1, TPD52 were higher in tumor tissues than in normal tissues, while KRT14, TF, STC2 were more highly expressed in normal breast tissues (**Figure 9A**). Then, we found that the protein expression of SLC16A2 was higher in BC tumor tissues, whereas that of BTG1 was higher in adjacent normal tissues by immunohistochemical staining assay (**Figure 9B**, **Supplementary Figure 6**).

## 4 Discussion

Breast cancer is a highly heterogeneous solid tumor, calling for individualized treatment for BC patients to improve prognosis. Though current surgery, endocrine, chemotherapy, and target therapy could improve the overall survival of BC patients, a large proportion of patients recur or progress, which leads to poor prognosis. Thus, investigating the differentially expressed genes and their roles in tumor malignant biological processes might help to analyze clinicopathological features of individual patients and offer precise therapeutic regimens and estimate outcomes for BC patients.

Hypoxia is one of the most impactful hallmarks of solid tumors that could influence tumor progression such as promoting tumor cell proliferation, invasion, and regulating cell cycle, energy metabolism, and immune escape (38–40). Recently, many studies have demonstrated that hypoxia status was considered as an important characteristic of the tumor microenvironment that has a close relationship with immune therapy sensitivity (41–43). Regarding breast cancer, hypoxia could induce cell growth by activating the glycogen metabolic program, improve migration, angiogenesis, and regulate apoptosis (44, 45). Moreover, hypoxia suppresses immune effector gene expression in immune cells, leading to immune effector cell dysfunction and resistance to anti-PD-1 therapy in triple-negative breast cancer (46).

Ferroptosis, a newly identified programmed cell death, has been found to have a relationship with tumor occurrence and development (47). Repression of ferroptosis could promote tumor progression, using anti-tumor drugs such as PI3K-AKT-mTOR pathway inhibitors (GDC-0941, MK-2206) which could promote sensitivity to ferroptosis in breast cancer cells (48). Some studies revealed that metformin could induce ferroptosis in breast cancer, which suggested that the patients simultaneously suffering from BC and type 2 diabetes could prolong their survival by metformin; however, further clinical trials were needed to provide more convincing evidence (49–51).

Despite the progress that has been made, the identification of effective prognostic biomarkers and the development of drugs targeting hypoxia and ferroptosis remain scarce. Antitumor therapeutic strategies directly targeting the hypoxic microenvironment are mostly focused on developing nanoparticles, and drugs targeting ferroptosis were far from clinical application (52). Reassuringly, recent studies have attracted our attention. It has been found that hypoxia can inhibit ferroptosis in hepatocellular carcinoma and breast cancer (53). Alternatively, ferroptosis could enhance the radiosensitivity of hypoxic tumor cells by amplifying oxidative stress or inhibiting antioxidant regulation (54). The nanoplatform-based tumor reoxygenation, which could generate the active superoxide radical ( ${\text{O}}_{2}^{-}$ ), together with H_2_O_2_-participated iron-involved Fenton reactions of ferroptosis, plays a synergistic role in overcoming hypoxia-induced chemotherapy resistance of osteosarcoma *in vivo* (55). These inspiring findings suggest that simultaneously suppressing hypoxia and inducing ferroptosis sensitivity of tumors may produce a potently synergistic antitumor effect.

Herein, we hypothesize that we could identify hypoxia and ferroptosis-related genes that associated with breast cancer prognosis, clinical characteristics, and immune microenvironment, to provide potential biomarkers and molecular targets for anti-tumor drug development and construct efficient gene signatures that could predict prognosis and simultaneously reflect tumor microenvironment characteristics as well as the ferroptosis status of breast cancer.

In this study, we included BC patients from the TCGA dataset and screened prognostic genes related to hypoxia and ferroptosis from known gene sets (MsigDB and ferroDB). The Lasso–Cox method was used to construct a predictive model (HFPS) based on these genes and divided BC patients into high- and low-risk groups. Notably, the high-risk group showed significantly worse overall survival, higher TNM stage, higher rate of lymph node invasion, and a lower rate of ER positive than the low risk group which could help us to implement individual strategies in clinical practice. The results were validated in the METABRIC BC cohort. Moreover, to predict the prognosis of patients, we constructed a nomogram model based on HFRS and prognostic clinical factors including age and TNM stage to predict 1-, 3-, 5-year overall survival of BC patients. The high predictive ability was validated by calibration curves, and ROC curves in both TCGA and METABRIC cohorts, which could help to make individual clinical decisions for patients.

Then we identified that there were more suppressive ferroptosis and higher hypoxia status in high-HFRS patients than in low-HFRS patients. In GSEA analysis, the key gene sets that assess tumor hypoxia status such as WINTER_HYPOXIA_METAGENE, WINTER_HYPOXIA_UP, HARRIS_HYPOXIA, and REACTOME_CELLULAR_RESPONSE_TO_HYPOXIA were highly enriched in patients with high HFRS. These results implied that BC patients with high HFRS were more likely to form hypoxia microenvironment. The mRNA expression of ferroptosis drivers were reduced in high HFRS group, while ferroptosis suppressors were highly expressed. Some evidence demonstrated that targeting ferroptosis-related genes and promoting ferroptosis sensitivity of tumor cells were promising approaches for reducing cancer progression (56). Thus, patients with a higher HFRS which also present a repressed ferroptosis status might profit from therapeutic drugs that could induce ferroptosis such as Lapatinib and Cisplatin (57–59).

By analyzing the immune microenvironment phenotype of the high- and low-HFRS groups, we found that the immune cell infiltration of B_cell_naive, T_cell_CD4, T_cell_CD8, and Tregs, macrohpage_M0, M2, monocytes, NK_cell_activated, and NK_cell_resting were significantly different between the high-and low-HFRS group by CIBERSORT analysis. In further ssGSEA analysis, we confirmed that HFRS score was negatively correlated with activated CD8 T cell and activated B cell, while it was positively correlated with MDSCs and Tregs. Studies have reported that repressed CD4, CD8 and T cell and activated B cells infiltration indicated an immune suppression microenvironment in cancer. MDSCs mediated immune suppression *via* expansion and differentiation of Tregs and limiting NKs, DCs, and the polarization of macrophages to M2-phenotype, and were associated with clinical outcome of BC (60). Tregs has been wildly reported to promote cancer immune escape and contribute to BC progression (61, 62). Studies have reported that tumor immune cell infiltration could be regulated by hypoxia status and related pathways. In breast cancer, hypoxia boosted CD8+ T cell infiltration in tumor tissue and increased sensitivity to immune checkpoint blockade (63). The CD4+ T cell has been wildly demonstrated to possess cytotoxic programs and can directly kill cancer cells (64). Hypoxia or HIF-1α signal pathway could influence CD4+ T cell function, metabolism, differentiation, and infiltration to enhance immunosuppression in tumors (65, 66). Suthen et al. reported that Tregs and immunosuppressive myeloid subsets were found to be significantly enriched in the hypoxia tumor tissue regions (67). Furthermore, patients in high the HFRS group showed significantly higher Stromal score and Immune score, while they have lower tumor purity in ESTIMATE analysis, which indicated that HFRS was associated with immune microenvironment of BC. In conclusion, high HFRS was positively correlated with immunosuppression in BC cancers.

Some of the HFRS genes have been demonstrated to serve as tumor oncogenes or tumor suppressors and might be correlated with tumor biological behavior and prognosis in diverse kinds of cancers. BTG1 has been reported as a tumor suppressor inhibiting tumor proliferation and migration and increasing anti-tumor therapy sensitivity in some kinds of tumors (68–70), including breast cancer (71–74). However, some studies reported that its overexpression promoted tumor malignancy in colorectal cancer (75). Researches demonstrated that STC2 could impair breast cancer cell growth, migration, and cell viability, which was consistent with our results (76, 77). In colorectal cancer, the upregulated STC2 was associated with a poorer prognosis (78). Lin et al. also reported that STC2 promoted pancreatic cancer migration, invasion, and EMT (79). A pan-cancer research found that STC2 was closely related to tumor immune microenvironment including immune cell infiltration, ICGs, MMRs, TMB, and MSI (80). One bioinformatic analysis reported that overexpression of CCT6A in tumor tissue was associated with poor breast cancer prognosis (81). Jie Jiang et al. reported that upregulated CCT6A in Ewing sarcoma was correlated with a worse prognosis (82). A similar result was observed in hepatocellular carcinoma (83). Studies of Bilandzic et al. implicated the basal epithelial marker KRT14 as an absolute determinant for ovarian cancer cells’ spheroid integrity, mesothelial attachment, invasive potential, and chemotherapy resistance, which could provide some *in vitro* evidences to explain the role KRT14 plays in cancer (84, 85). Thus, exploring the detail function of KRT14 in breast cancer is required in further studies. Recently, P4HA2 has been demonstrated to play important roles in tumor, but its function in cancers might be different. For example, P4HA2 induced EMT and promote tumor growth, migration, and invasion in cervical cancer and glioma (86, 87), while in prostate and pancreatic cancer, it served as a tumor suppressor (88, 89). Consistent with our findings, studies demonstrated that high P4HA2 expression was associated with poor survival in breast cancer (90, 91). PGK1 is a glycolytic enzyme that catalyzes the conversion of 1,3-diphosphoglycerate to 3-phosphoglycerate and participates in tumor angiogenesis (92, 93). Many studies reported PGK1 as a prognostic gene in cancers, and it has been demonstrated to promote EMT and the progression of breast cancer (94–99). SLC16A2 is a member of SLC16 gene family, that encodes monocarboxylate transporters, but its function in cancer has not been identified yet, which required further investigation (100). TF is also known as Transferrin which is essential for ferric iron transporting into cells and could influence iron metabolism in human, and might be involved in ferroptosis regulation in tumor cells indirectly (101). In addition, the knockdown of transferrin leads to decreased lapatinib-related BC cell death, but further *in vivo* experiments were absent (16). TPD52 is an oncogene and closely associated with prostate, breast cancer, and other cancers (102–104), which was consistent with our results. CISD1 reduces ferroptosis *via* iron-sulfur cluster biogenesis and was identified as prognostic ferroptosis-related genes in bladder cancer, lung cancer, and hepatocellular carcinoma (53, 105, 106). Although these researches provided some evidence to demonstrate its relation with tumor disease, its function in breast cancer has not been investigated yet. Thus, our study identified HFRS genes that might provide potential targets for the development of clinical therapeutic regimens.

Previous studies mainly focus on ferroptosis or hypoxia-related genes to develop prognostic models, but there is no study to consider the cell death regulation and microenvironment heterogeneity of breast cancer together. Our study firstly explored the effect of combining hypoxia and ferroptosis on breast cancer prognosis by constructing a novel predictive signature (HFRS) with hypoxia and ferroptosis-related genes. Additionally, HFRS could distinguish ferroptosis, hypoxia status, immune cell infiltration, and clinical characteristics of BC patients, which might help to make individual therapeutic strategies. Meanwhile, to improve the sensitivity and specificity of HFRS, we established a nomogram based on HFRS and clinical prognostic factors, which could also facilitate the clinical application of HFRS.

## Data availability statement

The original contributions presented in the study are included in the article/**Supplementary Material**. Further inquiries can be directed to the corresponding authors.

## Ethics statement

The study was reviewed and approved by Ethics committees of Wuhan Fourth Hospital. The patients/participants provided their written informed consent to participate in this study.

## Author contributions

JXZ and FL designed and constructed this study. XS, HY, and BW performed the data analysis, figure plotting, and writing. JJZ did the immunohistochemistry staining. FL, JW, and JS were responsible for the data acquisition and critical reading of the manuscript. All authors contributed to the article and approved the submitted version.
