# Supplementary figures and images for: Development and validation of a combined hypoxia and ferroptosis prognostic signature for breast cancer
Source: Front Oncol. 2023 Mar 14;13:1077342. doi: 10.3389/fonc.2023.1077342 (PMC10043308; doi:10.3389/fonc.2023.1077342)

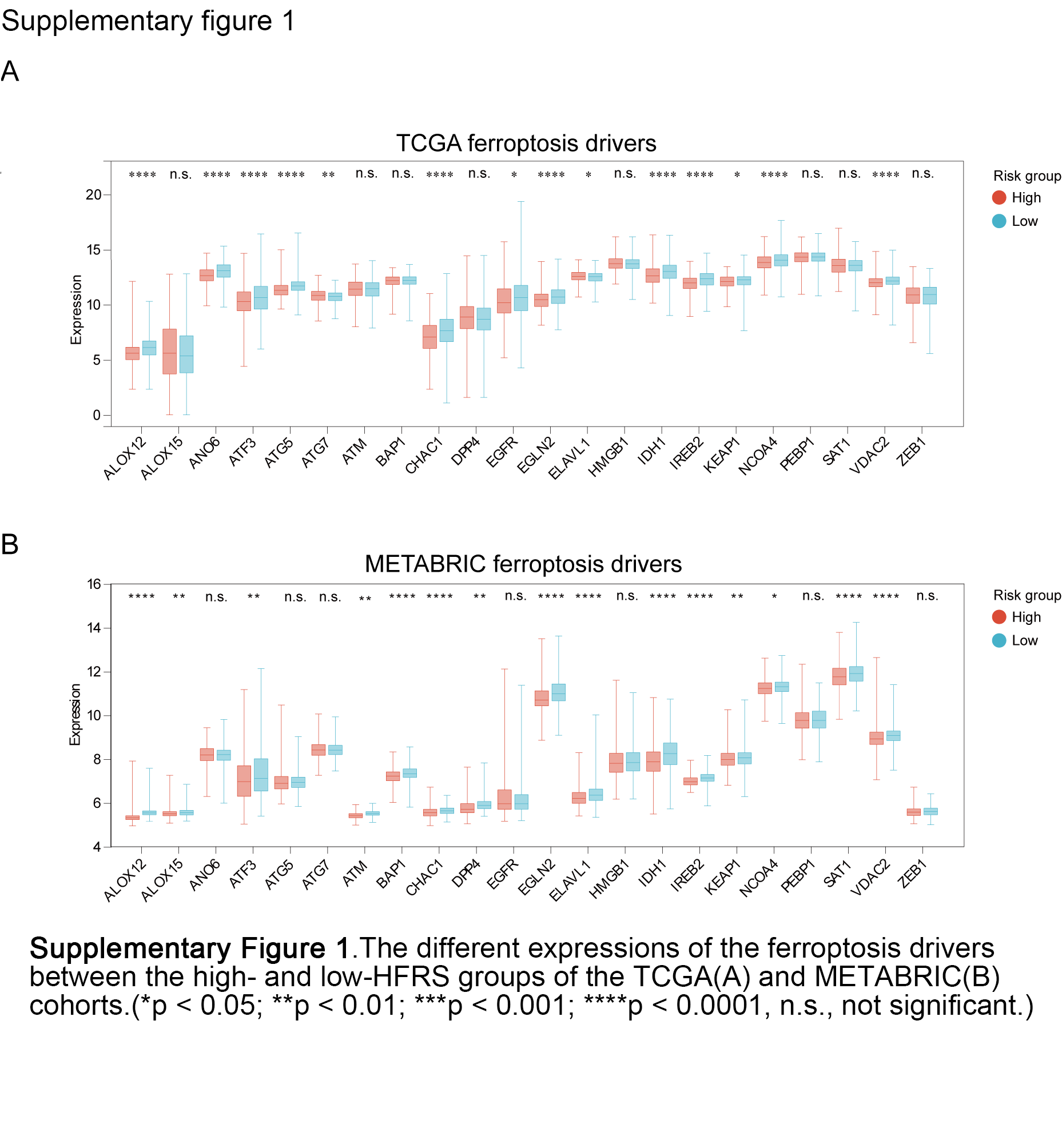

Supplement: Supplementary file 1 [file Image_1.tif]

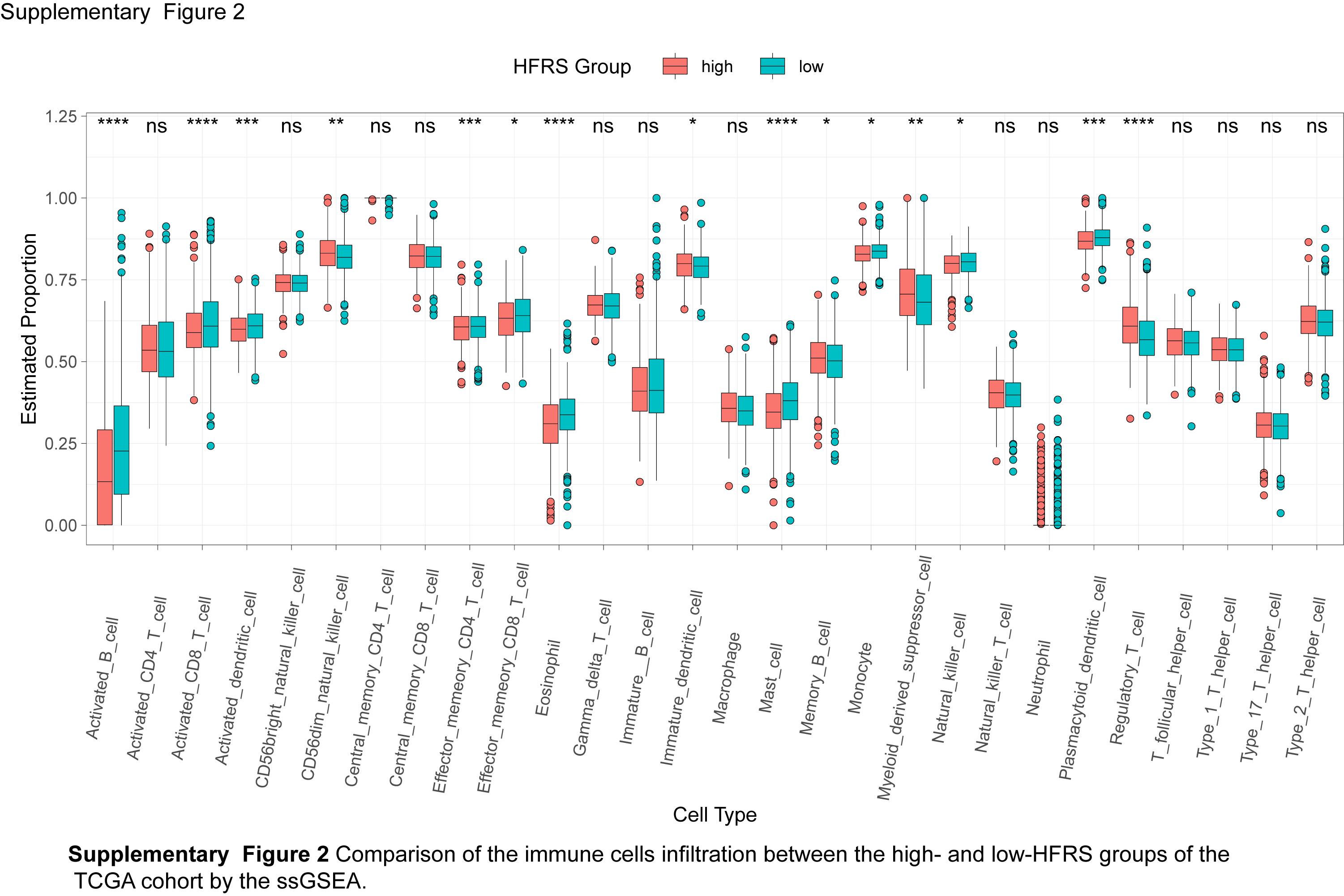

Supplement: Supplementary file 2 [file Image_2.tif]

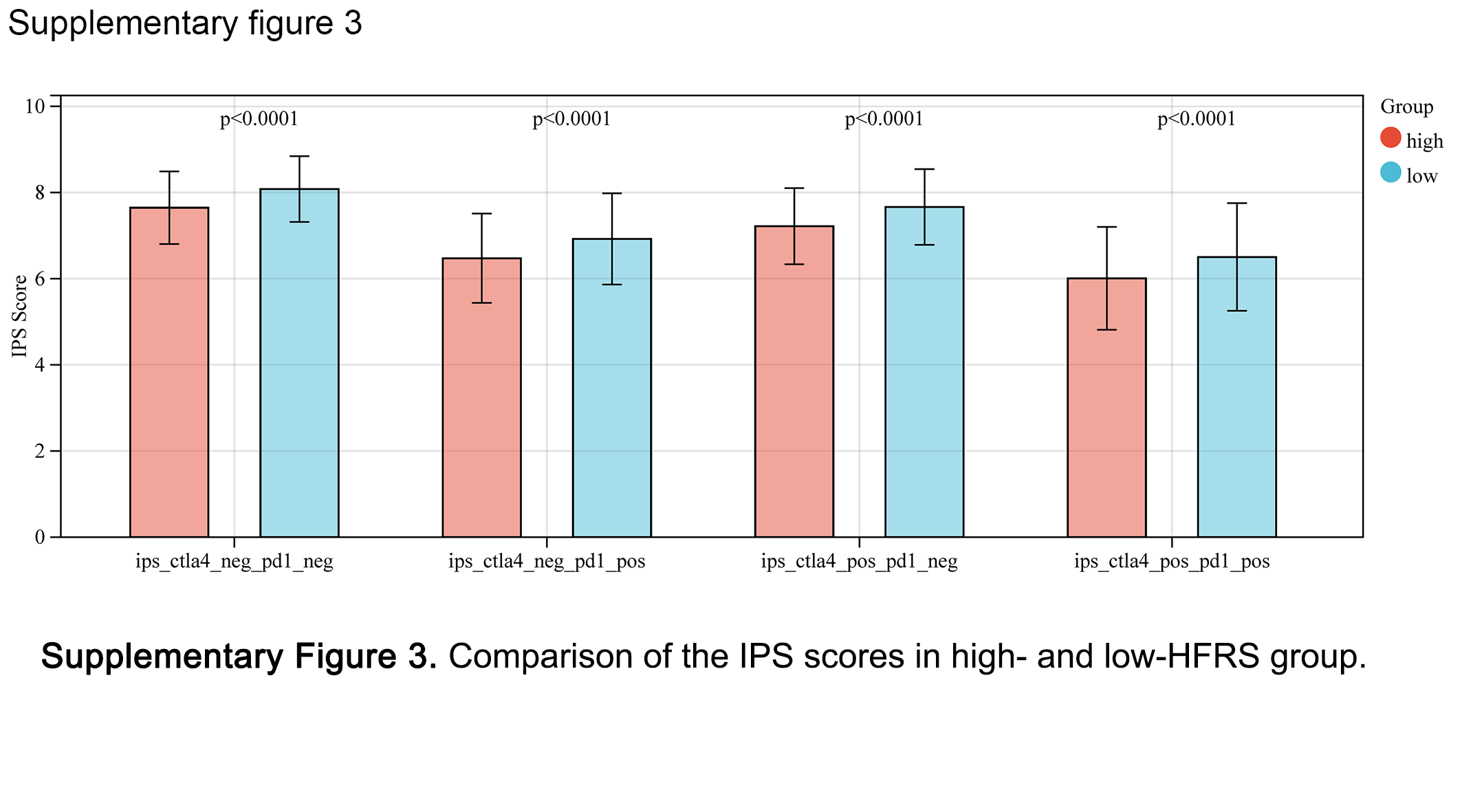

Supplement: Supplementary file 3 [file Image_3.tif]

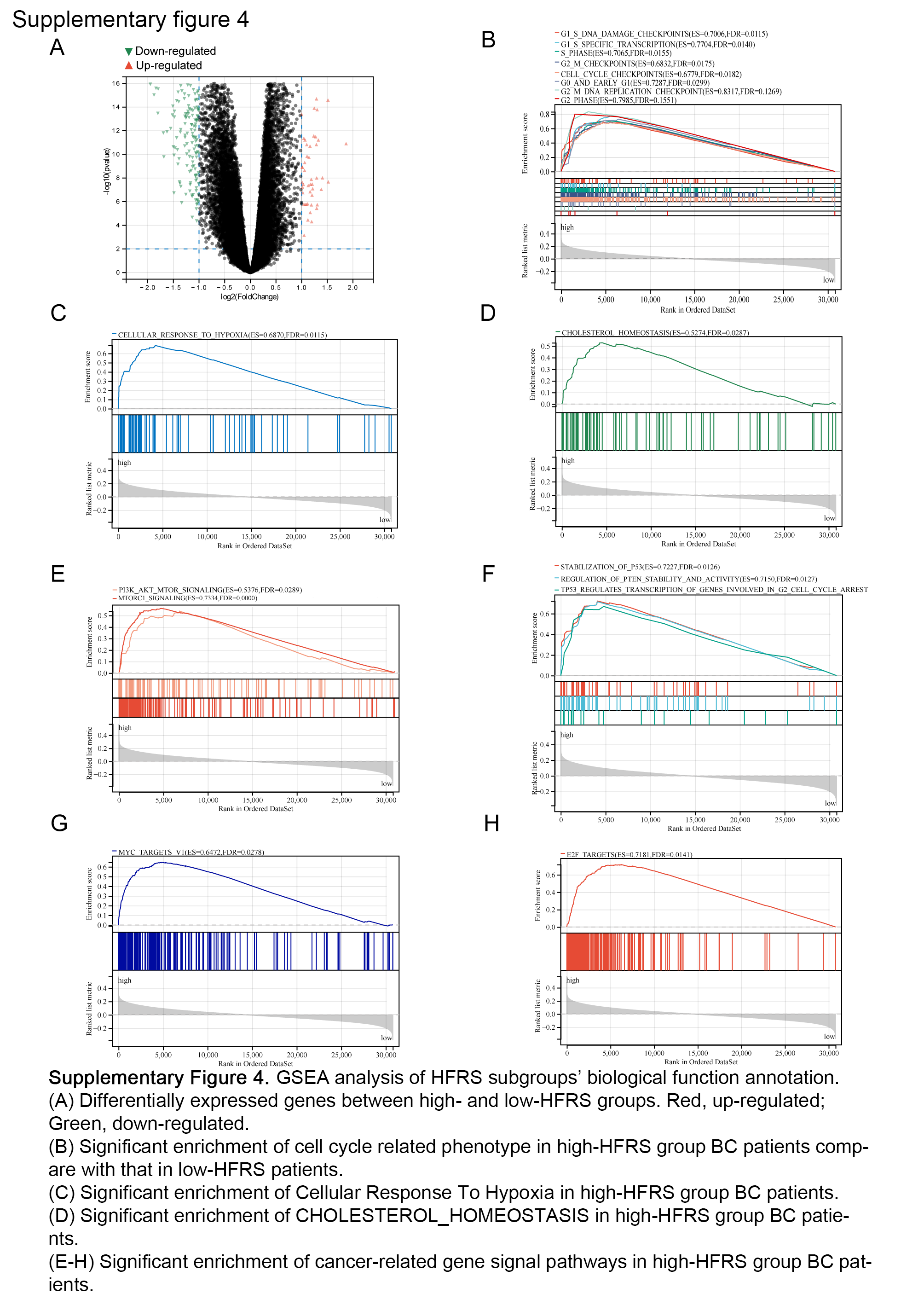

Supplement: Supplementary file 4 [file Image_4.tif]

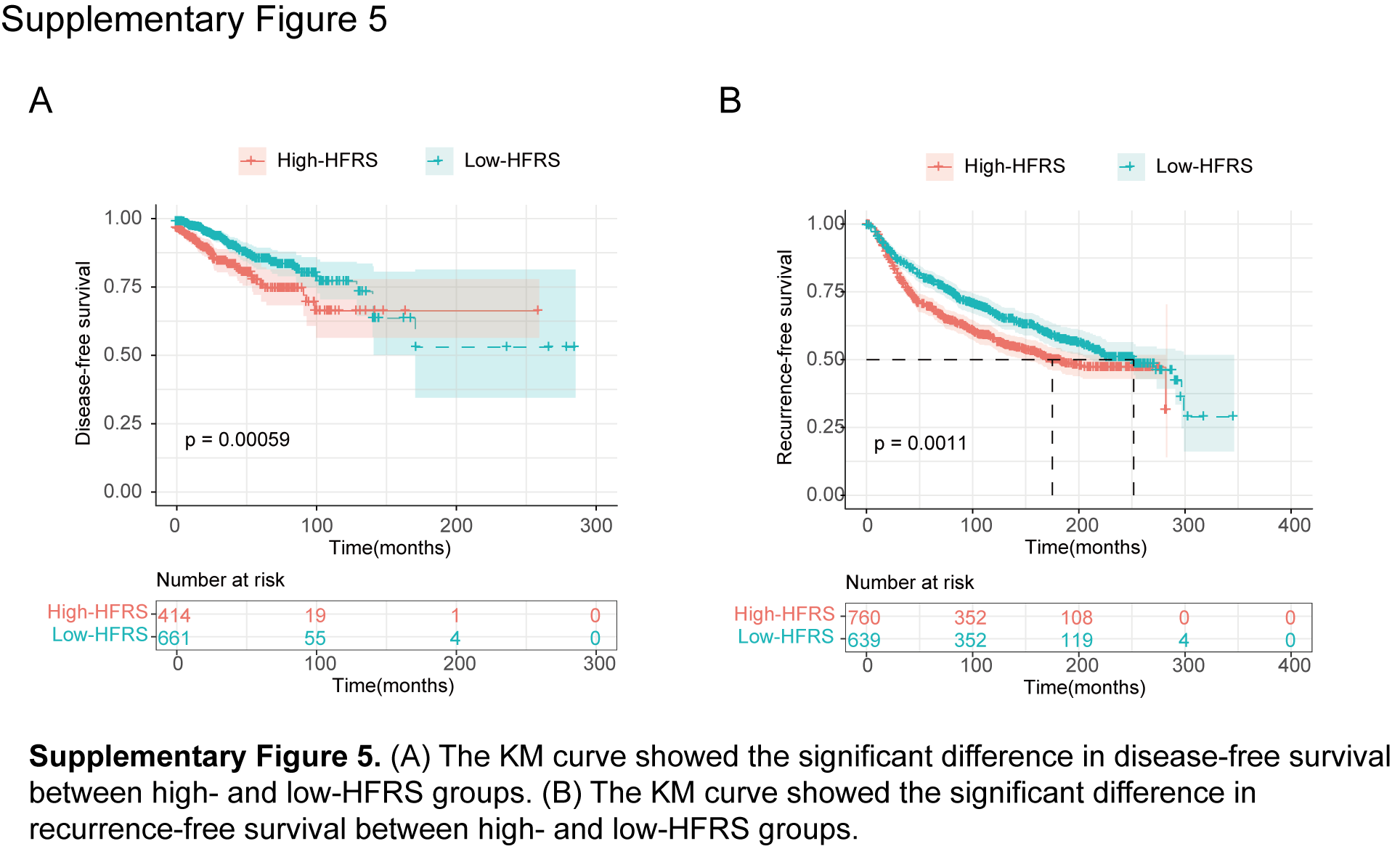

Supplement: Supplementary file 5 [file Image_5.tif]

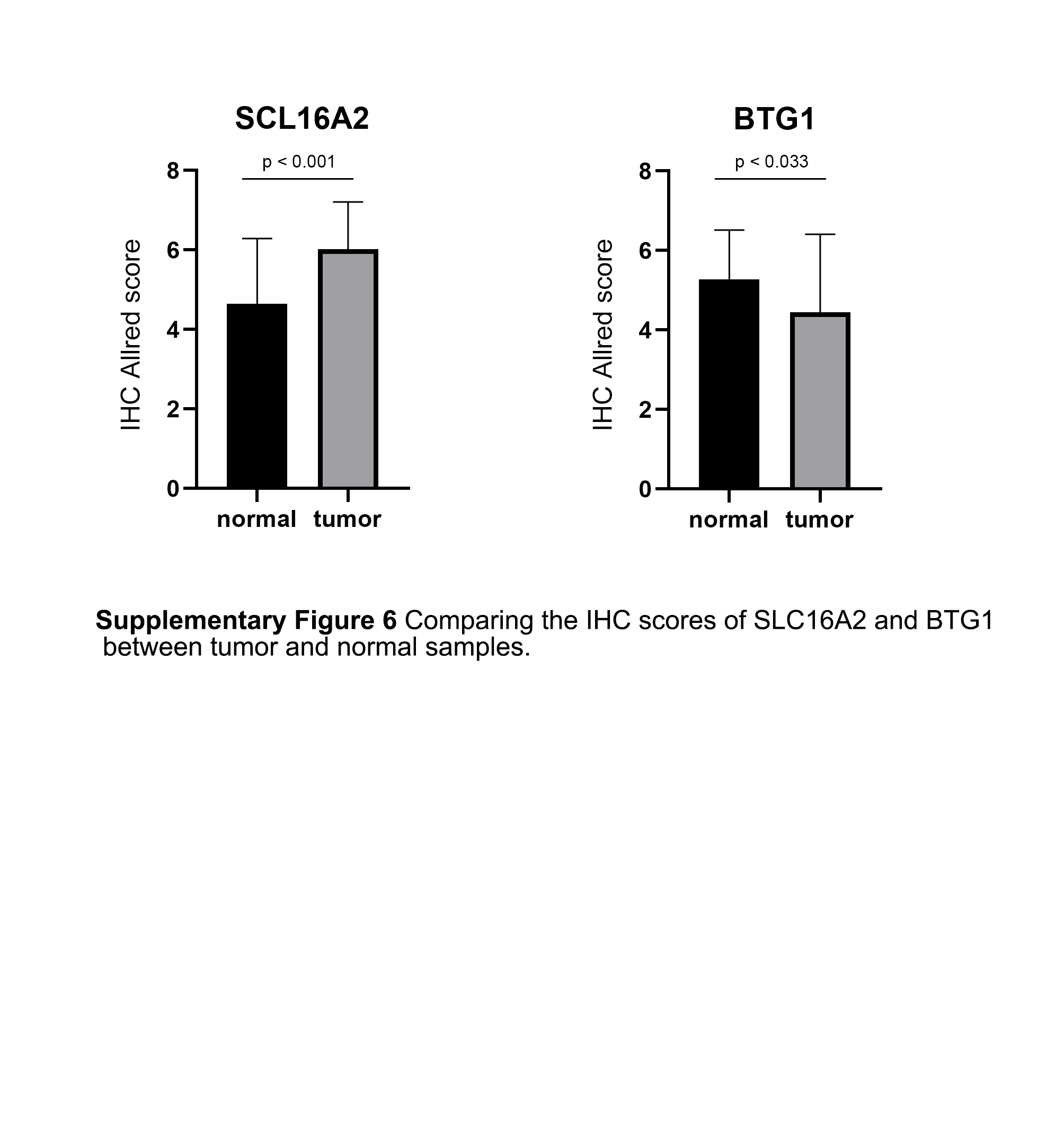

Supplement: Supplementary file 6 [file Image_6.tif]
