# Supplementary material for: Development and validation of a combined hypoxia and ferroptosis prognostic signature for breast cancer
Source: Front Oncol. 2023 Mar 14;13:1077342. doi: 10.3389/fonc.2023.1077342 (PMC10043308; doi:10.3389/fonc.2023.1077342)
Supplement: Supplementary file 7 [file Table_1.docx]

|  | **TCGA** | | **METABRIC** | |
| --- | --- | --- | --- | --- |
|  | High-HFRS | Low-HFRS | High-HFRS | Low-HFRS |
| Total sample | 414 | 661 | 760 | 639 |
| **Age**  ≤ median  > median | 197  217 | 355  306 | 374  331 | 317  273 |
| **Tumor stage**  I/II  III/IV  Unknown | 285  113  16 | 506  149  6 | 675  85  0 | 600  39  0 |
| **Histology types**  Infiltrating ductal carcinoma  Infiltrating lobular carcinoma  Others^b^  NA | 343  25  45  1 | 428  175  58  0 | 624  43  93  0 | 467  57  115  0 |
| **PAM50 subtype**  LumA  LumB  Her2  Basal  Normal | 39  217  83  74  1 | 205  276  31  122  27 | 161  341  134  109  15 | 286  215  42  67  29 |
| **Lymph node status**  Positive  Negative  NA | 187  145  82 | 284  308  69 | 396  364  0 | 253  386  0 |

**Supplementary Table 1 Clinicopathological features of BC cancers of TCGA and METABRIC cohorts**
